# Drilling Sequence Optimization Using Evolutionary Algorithms to Reduce Heat Accumulation for Femtosecond Laser Drilling with Multi-Spot Beam Profiles

**DOI:** 10.3390/ma16175775

**Published:** 2023-08-23

**Authors:** Christian Lutz, Jonas Helm, Katrin Tschirpke, Cemal Esen, Ralf Hellmann

**Affiliations:** 1 Applied Laser and Photonics Group, University of Applied Sciences Aschaffenburg, Würzburgerstraße 45, 63743 Aschaffenburg, Germany; 2 Applied Laser Technologies, Ruhr University Bochum, Universitätsstraße 150, 44801 Bochum, Germany

**Keywords:** USP laser processing, laser drilling, optimization of heat accumulation, evolutionary algorithms, thermal simulation

## Abstract

We report on laser drilling borehole arrays using ultrashort pulsed lasers with a particular focus on reducing the inadvertent heat accumulation across the workpiece by optimizing the drilling sequence. For the optimization, evolutionary algorithms are used and their results are verified by thermal simulation using Comsol and experimentally evaluated using a thermal imaging camera. To enhance process efficiency in terms of boreholes drilled per second, multi-spot approaches are employed using a spatial light modulator. However, as higher temperatures occur across the workpiece when using simultaneous multi-spot drilling as compared to a single-spot process, a subtle spatial distribution and sequence of the multi-spot approach has to be selected in order to limit the resulting local heat input over the processing time. Different optimization approaches based on evolutionary algorithms aid to select those drilling sequences which allow for the combination of a high efficiency of multi-spot profiles, a low-generated process temperature and a high-component quality. In particular, using a 4 × 4 laser spot array allows for the drilling of 40,000 boreholes in less than 76 s (526 boreholes/s) with a reduced temperature increase by about 35%, as compared to a single spot process when employing an optimized drilling sequence.

## 1. Introduction

Micro-material processing using ultrashort pulsed (USP) lasers is generally associated with a low-thermal input into the processed workpiece, i.e., a limited formation of a heat-affected zone, and is therefore considered with regard to cold laser ablation [1,2]. Nevertheless, when processing with high repetition rates and pulse energies, i.e., with increased average power of lasers nowadays available for industrial use, undesirable thermal effects occur, leading to heat accumulation in the processed component [3,4]. Especially in laser micro-drilling, such lasers are now used industrially to efficiently drill high numbers of micro-holes on short time scales [5,6,7,8]. The ensuing heat accumulation results in a low-component quality, being typically out of character for USP laser processes [9,10,11].

Especially for metal foils, heat accumulation can lead to distortion of the processed specimen, resulting in defocusing during the laser process, which in case of drilling processes, can lead to uneven drilling diameters or incomplete through holes. To investigate this process, we simulate the heat accumulation during the drilling of a high number of holes with a single spot or in a multi-spot mode. For the purpose of reducing heat accumulation by changing the drilling sequence, evolutionary algorithms are used to generate optimized drilling sequences according to different strategies. The obtained results are verified by comparing thermal simulations and experiments and are used in drilling processes to reduce the temperature level of the heat-affected zone, which results from a combination of laser parameters, material properties, and the ablation process.

Aiming to increase productivity, higher repetition rates and fluences are often used, which result in heat accumulation and plasma shielding effects [12,13,14,15,16]. While heat accumulation in components generally results in negative effects in the machining process, Gruner et al. [7] showed an increasing ablation rate using a high repetition rate of 48.78 MHz and a low fluence of 0.53 J/cm^2^ in a drilling process in 100 µm thick single-side polished silicon. Furthermore, without decreasing the resulting quality, a decreasing influence of the applied fluence for very high repetition rates and a decreasing ablation threshold were found as a result of heat accumulation. Due to the high relevance of heat accumulation for fast USP machining processes for industrial use, it is often investigated by simulations that represent the heating of the material surrounding the processing area [17,18,19,20,21]. Bornschlegel et al. specifically investigated the heat formation during the USP-laser ablation of stainless steel 1.4301 by measuring the workpiece temperature. By simulating the process with COMSOL Multiphysics, it was shown that the residual energy, which is below the ablation threshold of the Gaussian beam profile, does not contribute to the ablation process, but heats the surrounding material. In addition, the amount of residual energy that heats the surrounding material is shown to be larger the closer the applied fluence is to the ablation threshold of the material [22]. It can also be concluded that as long as the used fluence is similar to the calculated optimal range, which is around seven times the threshold fluence [23,24,25,26], a constant part of the heat input is an acceptable assumption. If the used fluence deviates significantly from this, non-uniform heating occurs during the process, so this assumption can no longer be made. While many simulations are based on the finite element method, which has a mesh with variable density that defines the resolution of the simulation [17,27], imulations can also be mesh free. An example of this is the Particle Strength Exchange method which is used in a multi-method comparative work by Afrasisabi et al. and yields high-resolution results [20].

The Traveling Salesman Problem is a well-known sequencing problem in optimization that takes into account intermediate destinations under certain constraints and thus tries to minimize the required travel time [28]. Although the sequence problem to be optimized in this work differs from a classical Traveling Salesman Problem, the applied solution strategies can be transferred to technical applications, e.g., in this study to reduce the inadvertent heat input into the workpiece during laser processing. Thus, heuristic algorithms to solve Traveling Salesman Problems can be used to optimize or decrease the running time of drill hole arrays drilled by CNC machines [29,30]. Genetic algorithms, which also belong to the class of heuristic algorithms, are still used today as an effective method for solving sequence problems with the Lin–Kernighan–Helsgaun algorithm being widely used since its publication in 1973 [31].

Since tangible optimization problems often do not have an analytical solution, numerical methods, including genetic algorithms, offer the possibility of an approximate solution to the optimization. The genetic algorithm, first introduced by Holland et al. [32], offers the advantage of simple implementation for solving practical optimization problems such as, trunk lid mechanisms for sedan vehicles [33], a five-bar planar manipulator [34] and four-bar linkage systems [35]; genetic algorithms are also used for model fitting in the field of cancer chemotherapy [36]. In addition to the use of genetic algorithms, particle swarm optimization [37], differential evolution [38], ant colony optimization [39], artificial bee colony [40] and several others, are also important optimization methods [33]. 

## 2. Experimental

We employ a Yb:YAG USP laser (Amplitude Tangor, Amplitude Systems, Bordeaux, France) with a maximum output power of 100 W at an infrared emission at 1030 nm and a variable repetition rate between single shot and 40 MHz. The laser, with a tunable pulse length between 800 fs and 10 ps, is integrated into a micromachining system (RDX-1000 FBS, Pulsar Photonics GmbH, Herzogenrath, Germany), which uses a galvo scanner (IntelliSCANse14, SCANLAB GmbH, Pucheim, Germany) and a F-Theta lens (LINOS F-Theta Ronar, QiOptiq Photonics GmbH and Co. KG, Göttingen, Germany) with a focal length of 100 mm. The with a high-resolution CCD camera (UI149xLE, IDS Imaging Development Systems GmbH, Obersulm, Germany) measured focal diameter ${\;d}_{o}$ is 41 µm $\left(1/e^{2}\right)$. With $E_{p}$ being the pulse energy and $r_{0}$ the radius of the focal beam, the resulting fluence is calculated according $\Phi =E_{p}/\left(\pi \cdot r_{0}^{2}\right)$. Throughout the experiments explained in Section 4, a repetition rate of 50 kHz was used to drill the holes. A spatial light modulator (SLM)-based beam-shaping module allows dynamic beam shaping to generate different spot distributions with a maximum frame rate of 60 Hz. To protect the LCOS-SLM (X1522, Hamamatsu Photonics, Shizuoka, Japan), an effective active liquid cooling system was integrated.

**Figure 1:**
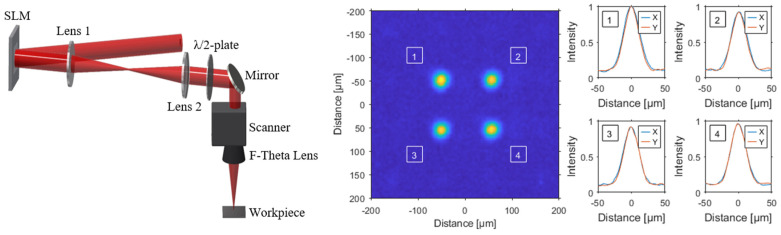
Schematic illustration of the setup for femtosecond laser drilling using a spatial light modulator (SLM) and a 4f-telescope with a galvo scanner including an F-Theta lens. Also shown are measured intensity profiles of the 2 × 2 beam splitter with a separation of 100 µm.

Based on previously reported drilling results, a repetition rate of 50 kHz was used in the following drilling experiments [25]. In addition, the shortest possible pulse length of the employed laser system of 800 fs was used in order to minimize objectionable thermal impacts and to achieve the highest possible borehole quality for both, single spot and a multi-spot (2 × 2 and 4 × 4) beam splitters with a separation between the spots of 100 µm. The beam profile, including the intensity distribution of the 2 × 2 spot profiles, is shown in Figure 1. The required computer-generated holograms for the SLM are calculated using an iterative Fourier transform algorithm. After the initial calculation of the hologram, the uniformity of the single sub-beams in the focal plane is further improved by a feedback-loop-based weighted Gerchberg–Saxton algorithm [41]. Thus, for the four spot profile, a uniformity of 0.98 can be achieved, as shown in Figure 1.

An infrared camera (thermoIMAGER TIM640, Micro-Epsilon Messtechnik GmbH and Co. KG, Ortenburg, Germany) is used to perform temperature measurements during the drilling process. The camera allows imaging a temperature range between −20 °C and 1500 °C at a spatial resolution of 640 × 480 pixels with a maximum image recording rate of 32 Hz. As the low-emission coefficient of stainless steel may lead to falsification of the thermal measurement results, the steel sheet was thinly coated with a 7 µm layer of a special camera lacquer in order to improve the quality of the temperature measurement by suppressing the ambient radiation.

Due to the frequent use of stainless steel, such as for microfilters [42], injection nozzles [43] and surfaces with altered wettability properties [44], stainless steel foil (X5CrNi18-10) with a thickness of 50 µm is used in this drilling study. For the thermal simulation, relevant thermo-physical properties are taken from the literature [45]. The reflectivity of the specific material on hand was yet to be determined experimentally for an angle of incidence of 90°. A spectrometer (Spectro 320, Instrument Systems GmbH, Munich, Germany) with a spectral range from 190 nm to 2500 nm and a resolution of 0.1 nm was used to perform the reflectivity measurements. To reproduce the machining situation in the drilling process, an equipped goniometer with an angular resolution of 0.1° was set an angle of 90°. For the applied laser wavelength of 1030 nm, the material showed a reflectivity of 67.5%.

## 3. Simulation and Optimization

The thermal simulation of the drilling process is based on COMSOL Multiphysics 5.6 software (Comsol Multiphysics GmbH, Goettingen, Germany), which calculates the heat accumulation in calculation steps of 5 ms. The heat distribution across the laser-processed stainless steel sheet was simulated on time scales of the experimental drilling process with the maximum thermography camera frame rate of 32 Hz, i.e., in a steady state, disregarding excitation and interaction effects on ultrashort time scales to achieve the highest possible comparability. The infrared camera measures the effective temperature generated across the component, which allows working with an appropriate correction factor for comparison in the simulation. Bornschlegel et al. [22] extensively investigated heat accumulation by USP laser processing in stainless steel for different fluence ranges and repetition rates using both simulations and experiments. For processes close to the used optimal fluence, here, the experimental data, the temperature level and temperature profiles of the simulation with a residual heat input factor of 0.3 are in good agreement, similar to the analysis of Bauer et al. [4].

**Figure 2:**
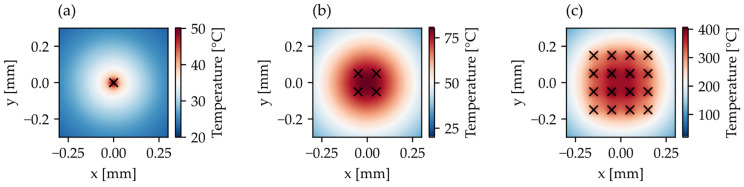
Simulation results of heat distribution in 50 µm thick stainless steel sheet after 20 ms by a static processing through a beam profile with one (**a**), 4 (**b**) and 16 (**c**) spots with a fluence of 0.8 J/cm^2^ (0.575 W per Spot). The 2 × 2 and 4 × 4 beam splitters have a separation of 100 µm.

Figure 2 shows the simulation result of a static processing disregarding ablation after 20 ms using a single spot (Figure 2a) and a 2 × 2 (Figure 2b) or a 4 × 4 beam splitter (Figure 2c), respectively. The fluence per spot is 0.8 J/cm^2^, thus the applied average power is increased proportionally to the number of spots. While a single spot results in a temperature of approx. 50 °C in the immediate area 250 µm surrounding the borehole, the use of a 2 × 2 or 4 × 4 beam splitter with the corresponding power applied results in a temperature of approx. 80 °C or 400 °C, respectively. While the heat distribution of the single spot (Figure 2a), as well as the 2 × 2 beam splitter (Figure 2b), resemble a circular distribution, the use of the larger 4 × 4 beam splitter (Figure 2c) leads to a barrel-type distortion of the heat distribution.

A genetic algorithm was used to find an optimal drilling sequence that minimizes heat accumulation. Genetic algorithms are particularly well suited for determining optimal sequences. First, a certain number of admissible solutions—the start generation—was generated. The quality of the solutions was evaluated with a fitness function. In further steps, mutation and recombination were used to compute new generations from this start generation, which have a better value with respect to the fitness function.

The algorithm itself was implemented in Python because the DEAP library provides efficient programs for implementing genetic algorithms. The DEAP library produces evolutionary algorithms via arbitrarily combinable genetic operators and selection operators, and permits the arbitrary stringing of functions. In addition to supporting genetic algorithms and evolution strategies, DEAP also supports particle swarm optimization, differential evolution and algorithms for estimating distributions [46,47,48].

**Figure 3:**
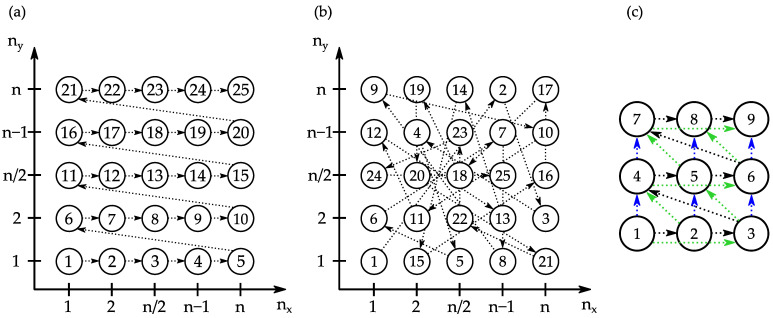
Schematic illustration of a borehole array with $n_{x}\times n_{y}$ holes which is drilled with the standard sequence (**a**) and a exemplary optimized drilling sequence (**b**). (**c**) shows the distances from one hole to the next, and the next but one hole, which are considered in the optimization.

For the success of a genetic algorithm, it is crucial to find a fitness function that reflects the real behavior as close as possible. Since a distance between two successive drillings is to be assumed as particularly critical, the minimum distance is computed after conversion of the drilling sequences into Cartesian coordinates. Different fitness functions were tested based on a list of distances. In all strategies, only the spatial distances between successive boreholes were considered. First, the minimum distance between two consecutive boreholes should be maximized (S1), and second, the mean distance between two consecutive boreholes (S2). The third strategy (S3) is a combination of the first two strategies. The results were compared with the standard sequence, which is shown in Figure 3a.

In addition to the distances between successive boreholes, the distance between the individual boreholes to the respective borehole, which is drilled two and three drilling processes later, can also be considered in order to prevent continuous jumping back and forth. For each sequence, a list with the corresponding distances was calculated. In addition, when considering many distances per sequence, more generations are needed to optimize the quality value. For these reasons, only the distances between each hole to the next, and the next but one hole are considered to keep the optimization time as short as possible. In addition, it is important to avoid, if possible, the boreholes that are drilled in a short time window, which extend over three times the drilling time, drilled in a small spatial area within the borehole array.

The algorithm creates 100,000 generations. Only for the very extended examples with 200 × 200 drilled boreholes, the number was reduced to 10,000. The A µ + λ strategy was implemented, which means that both members of the parent generation and those of the offspring generation are available for the selection of the members with the best fitness function value. In this context, µ corresponds to the number of members of the parent generation and λ to the number of descendants. While each individual is a whole drilling sequence, the population thus initially consists of µ sequences, generating λ descendants, so from µ + λ sequences the best µ’s are chosen for the following generation. A probability of random mutation of 30% for the drilling sequence was chosen as a balance between the highest possible quality value and an acceptable calculation time. While a lower percentage for the random mutation results in a lower calculation time for a constant number of generations, at the same time, more generations are needed to reach a certain quality value.

**Figure 4:**
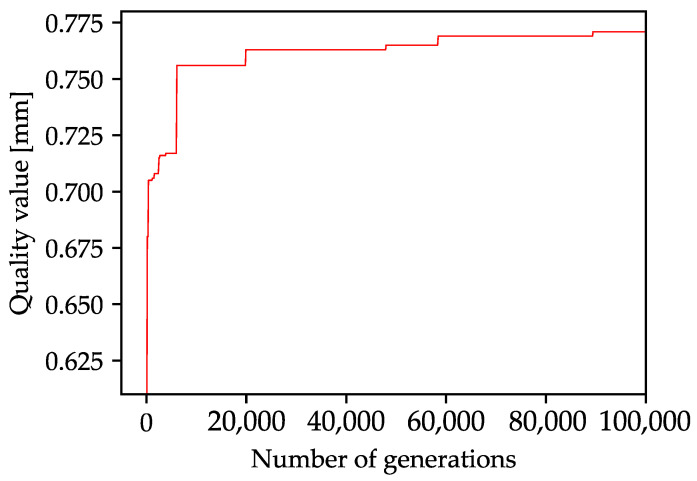
Progression plot of the quality value in terms of the minimum borehole distance during the optimization of a 40 × 40 array drilled with a 4 × 4 beam splitter over 100,000 generations.

The optimization runtime depends significantly on the length of the list of borehole distances, which, as explained before, depends on the dimension of the array as well as on the considered spectrum of successive boreholes. In addition, the number of optimization generations is a critical factor, which is why a compromise must always be made between the highest possible quality value and the shortest possible calculation time. Due to the different array sizes and the number of optimization generations, the optimization time (considering the next and the next but one hole) ranges from a few minutes for a 10 × 10 array with 100,000 generations to about 50 h for a 200 × 200 array with 10,000 generations. In Figure 4, the development of the quality value during the calculation of a 40 × 40 array calculated with 100,000 generations is shown. Apparently, the greatest optimization of minimum borehole spacing occurs in the first 10,000 generations, after which only minor improvements are achieved until the 100,000 generations are reached. The progression of the quality value, with a higher improvement gradient at the beginning of the optimization and a subsequent saturation of the function, is typical for optimizations using genetic algorithms, and can also be observed in other studies [33,35,49].

The result after completion of the optimization is a drilling sequence that is converted into Cartesian coordinates for use in the simulation environment, as well as on the laser machine. An optimized drilling strategy can result in a higher overall process time depending on the array size and due to changed distances between the drilled holes. To ensure optimal comparability, a corresponding delay is implemented between each drilling position in the standard drilling sequence.

## 4. Results

**Figure 5:**
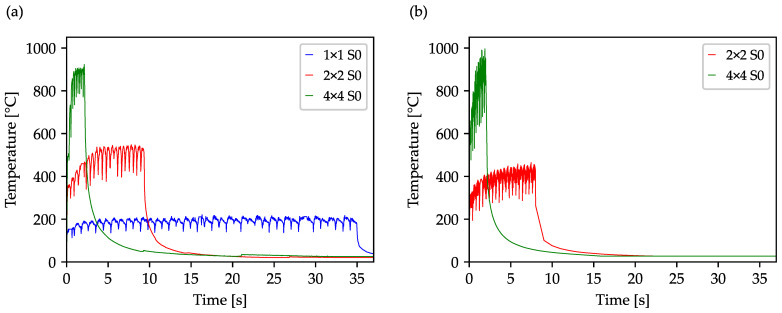
Comparison of the experimental (**a**) and simulated (**b**) determined temperature curves for a 40 × 40 array with a separation of 100 µm. The holes were drilled with different spot profiles for a fluence of 0.8 J/cm^2^ (each spot) and a drilling time of 0.02 s (per bore hole).

To obtain the acquainted to the heat accumulation during the drilling process, and to establish a basis for comparison, the first step was to drill a 40 × 40 array using a single spot as well as with a 2 × 2 and 4 × 4 multi-spot profile using the standard sequence shown in Figure 3a. The laser power used in the process is based on a fluence of 0.8 J/cm^2^ per spot and varies with the number of spots. As shown in Figure 5a, the drilling time of the 1600 holes was significantly reduced by using multi-spot beam profiles. The undulated variation of the temperature development reveals the line-by-line processing strategy of the drill array (cf. Figure 3a), leading to an undulated varying component temperature. Moreover, using a higher number of spots results in an overall temperature increase. While the maximum temperature in the single-spot is about 200 °C, temperatures of about 550 °C and 920 °C were reached when 4 or 16 spots are used. This is assigned to the increasing average power employed in the respective machining process. Thus, it can be concluded that the use of 4 or 16 times higher power leads to a machining temperature that is 2.6 or 4.6 times higher, i.e., the relationship between the increasing spot number (thus productivity) and temperature increase does not follow a linear behavior. Despite the higher component temperature however, the use of multi-spots to increase the overall productivity of the drilling process is highly reasonable, which is why this will be pursued in the following experiments. 

To verify the experimentally determined temperature profiles and to evaluate the simulation, the next step was to simulate the standard sequence using the 2 × 2 and 4 × 4 multi-spot profile without the use of optimization, which is shown in Figure 5b. Obviously, both the attained temperature range of the different used beam profiles and the overall temperature profile in both simulation and experiment are in good agreement. Remaining quantitative differences on the order of 70–80 °C are attributed to the lower sampling rate and spatial resolution of the thermal imaging camera as compared to the simulation, thus occurring maxima may not be entirely recorded. The periodic heating phases due to the drilling of each line and the subsequent jump, as well as the cooling behavior to be expected according to Newton’s cooling law after the process, are clearly seen and concordant. The typically undulated, wavelike character is also shown, whereby the short drop of the maximum measured temperature in the area of the inflection point between the oscillations results from the jump from the last hole of a drilled row to the first hole of a new row. Please note that the finely displayed profile of the simulated temperature graphs results from significantly smaller calculation steps as compared to the frame rate of the thermal imaging camera. The high agreement of the results from simulation and experiment allows the validation of the drilling process so that this can be used to demonstrate the effects of changing the drilling sequence. 

**Figure 6:**
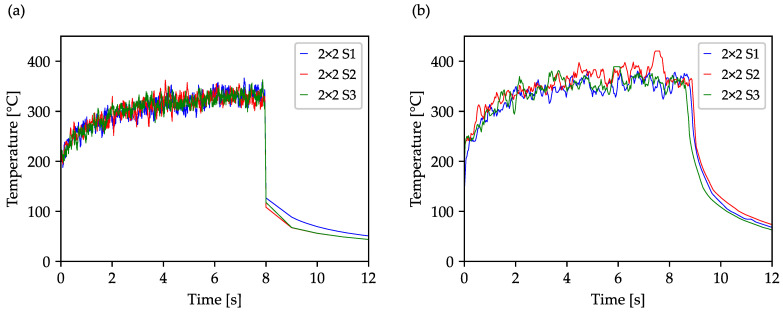
Simulated (**a**) and experimentally measured (**b**) temperature plots of the three investigated strategies S1, S2 and S3. The plots of a 40 × 40 array with a separation of 100 µm were drilled using a 2 × 2 beam splitter with an average power of 2.3 W and a repetition rate of 50 kHz.

As described in Section 3, the optimization of the drilling sequence was performed using three different target functions. Firstly, the minimum distance between two consecutive boreholes should be maximized (S1), secondly, the mean distance between two consecutive boreholes (S2), and thirdly, the combination of the first two strategies (S3). To evaluate the changed drilling sequences and their effect on the heat accumulation across the workpiece, the optimization result was transferred into a list of Cartesian coordinates and used in the simulation and via the laser system, based on an array of 40 × 40 drill holes. Here, again, the 2 × 2 multi-spot array was used to achieve a correspondingly shorter process time. The results are summarized in Figure 6. The simulated temperature across the workpiece differs only marginally, yet remains dependent on the used strategy. Overall, the temperature profile during the drilling process, the maximum reached temperature and the cooling behavior in the component are almost the same. The average temperature within the drilled area starts at about 200 °C and increases gradually, reaching about 350 °C at the end of the drilling process. 

The experimental realization of the drilling study to compare the optimization strategies also shows a very similar behavior. Additionally, in this experiment, the good agreement of the temperatures achieved between simulation and experiment are found. The undulated, wavelike temperature evolvement, resulting from the row-by-row drilling of the standard sequence, no longer occurs in the optimized sequences in both diagrams (Figure 6a,b). Only the lower temporal resolution of the thermal imaging camera compared to the simulation leads to fewer data points in the individual graphs, which is reflected in a lower density of temperature jumps. Due to the small temperature differences between the investigated strategies S1, S2 and S3, only the S1 strategy is further pursued in the following investigations.

**Figure 7:**
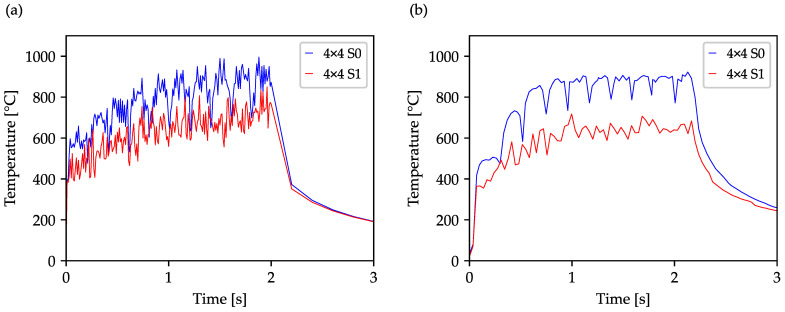
Comsol simulated (**a**) and experimentally measured (**b**) temperature plots of a 40 × 40 array drilled with a 4 × 4 beam splitter using the standard strategy and the optimized strategy S1. In the 0.02 s drilling process, an average power of 9.2 W and a repetition rate of 50 kHz were used, corresponding to a fluence of 0.8 J/cm^2^ in the single spot.

In the next step, the influence on the heat accumulation and the resulting component temperature were compared by the standard strategy (S0) with the optimized drilling sequence S1 using a 40 × 40 array drilled with a 4 × 4 beam splitter. The resulting temperature plots originating from the simulation and experiment are shown in Figure 7, showing that using an average power of 9.2 W for 16 spots results in temperatures about twice as high as those shown in Figure 6 using a 2 × 2 beam splitter and S1. Drilling in a series, using standard strategy leads to a wave-like temperature, rises to a maximum of about 996 °C in the simulation and 923 °C in the experiment. By optimizing the drilling sequence, the undulated progression is interrupted by the jump to spatially more distant boreholes. The greater distance between the following boreholes leads to a better distribution of the introduced heat and thus to a lower maximum temperature of 849 °C in the simulation and 718 °C in the experiment. Considering the Celsius scale-based average temperature, the optimization of the drilling sequence leads to a reduction of 150 °C in the simulation and 205 °C in the experiment, which corresponds to, respectively, a percentage of 19% and 26%. The significant reduction of the temperature highlights the beneficial influence of the drilling sequence optimization and represents a benefit for the generation of high-quality borehole arrays.

**Figure 8:**
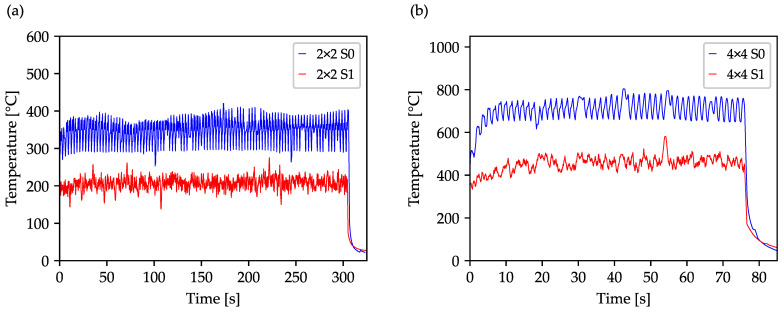
Temperature plots of a 200 × 200 array with a borehole separation of 100 µm measured experimentally with a thermal imaging camera and drilled with a 2 × 2 (**a**) and 4 × 4 (**b**) beam profile with an average power of 2.3 W and 9.2 W, respectively. The drilling process was performed using the optimized S1 sequence with a repetition rate of 50 kHz in addition to the standard drilling sequence.

In industrial practice, larger perforated surfaces are often used for the production of micro filters, which also require an even higher number of drilled holes. Opposite to our previous study on optimizing drilling sequences using a Simplex Algorithm [25], here we employ an optimization based on evolutionary algorithms and thus focus on the optimization of larger borehole arrays, which could not be optimized in an acceptable time with the previously used algorithms. Therefore, Figure 8 depicts the experimentally determined temperature curves of a 200 × 200 array with a separation of 100 µm. For comparison, the 40,000 drilled holes were generated with a 2 × 2 (Figure 8a) and 4 × 4 beam splitter (Figure 8b) using standard sequence and optimized strategy S1. Due to the significantly larger surface area to be machined, longer travel distances between the hole positions result in a process delay, which is why the total process time for manufacturing the array is disproportionately longer as compared to the 40 × 40 array shown in Figure 7. Considering the graphs generated by 4 × 4 beam splitters, again a reduction of the average temperature is shown, in this case by 252 °C, which corresponds to a reduction of about 35.7% based on the achieved average temperatures without sequence optimization on the Celsius scale. It is also noticeable that the maximum temperatures are slightly lower than the 40 × 40 array shown in Figure 7, which could be due to the overall larger component size and thus a larger spatial distribution of heat. The use of the 2 × 2 beam splitter, and thus a lower average power of 2.3 W, extends the drilling time of the 200 × 200 array to approximately 310 s. However, even within this longer drilling time, a significant reduction of 141 °C or 40.5% on average is achieved, which represents a maximum in the performed study.

## 5. Conclusions

Evolutionary algorithms, thermal simulations and experimental investigations were used in this study to investigate and reduce heat accumulation during drilling of borehole arrays of up to 40,000 (200 × 200) holes using a femtosecond laser in metal sheets. A genetic algorithm with up to 100,000 generations was used to optimize the drilling sequences, which was controlled by a quality value in terms of the minimum borehole spacing. By selectively optimizing the drilling sequence using different strategies, the process temperature, using a single spot as well as beam profiles with 2 × 2 and 4 × 4 beam splitters, was reduced up to 40.5% compared to average temperatures with the standard strategy on the Celsius scale. The significant reduction of the process temperature by optimizing the drilling sequence enables a lower thermal load on the material in future drilling processes of thousands of holes, which is characterized by a lower thermal distortion and smaller heat-affected zones. As the perceptual reduction of the average temperature is higher for large borehole arrays than for smaller ones, the presented method also paves the way towards highly efficient industrial drilling processes.
